# Comparison of videolaryngoscope-guided versus standard digital insertion techniques of the ProSeal™ laryngeal mask airway: a prospective randomized study

**DOI:** 10.1186/s12871-019-0915-3

**Published:** 2019-12-30

**Authors:** Ulku Ozgul, Feray Akgul Erdil, Mehmet Ali Erdogan, Zekine Begec, Cemil Colak, Aytac Yucel, Mahmut Durmus

**Affiliations:** 1 School of Medicine, Department of Anesthesiology and Reanimation, Inonu University, Malatya, Turkey; 2 School of Medicine, Department of Biostatistics, and Medical Informatics, Inonu University, Malatya, Turkey

**Keywords:** Equipment, Proseal laryngeal mask airway, Insertion technique, Video-laryngoscopy

## Abstract

**Background:**

This study were designed to investigate the usefulness of the videolaryngoscope-guided insertion technique compared with the standard digital technique for the insertion success rate and insertion conditions of the Proseal™ laryngeal mask airway (PLMA).

**Methods:**

Prospective, one hundred and nineteen patients (ASA I–II, aged 18–65 yr) were randomly divided for PLMA insertion using the videolaryngoscope-guided technique or the standard digital technique. The PLMA was inserted according to the manufacturer’s instructions in the standard digital technique group. The videolaryngoscope-guided technique was performed a C-MAC® videolaryngoscope with D-Blade, under gentle videolaryngoscope guidance, the epiglottis was lifted, and the PLMA was advanced until the tip of the distal cuff reached the oesophagus inlet. The number of insertion attempts, insertion time, oropharyngeal leak pressure, leak volume, fiberoptic bronchoscopic view, peak inspiratory pressure, ease of gastric tube placement, hemodynamic changes, visible blood on PLMA and postoperative airway morbidity were recorded.

**Results:**

The first-attempt success rate (the primary outcome) was higher in the videolaryngoscope-guided technique than in the standard digital technique (*p* = 0.029). The effect size values with 95% confidence interval were 0.19 (0.01–0.36) for the first and second attempts, 0.09 (− 0.08–0.27) for the first and third attempts, and not computed for the second and third attempts by the groups, respectively.

**Conclusion:**

Videolaryngoscope-guided insertion technique can be a help in case of difficult positioning of a PLMA and can improve the PLMA performance in some conditions. We suggest that the videolaryngoscope-guided technique may be a useful technique if the digital technique fails**.**

**Trial registration:**

ClinicalTrials.gov NCT03852589 date of registration: February 22th 2019.

## Background

The ProSeal™ laryngeal mask airway (PLMA; Teleflex Medical Athlone, Co. Westmeath, Ireland) is a laryngeal mask device with a double cuff to improve the seal and incorporates a drainage tube to prevent a risk of aspiration and gastric insufflation. The PLMA is inserted via digital manipulation, similar to how the classic™ laryngeal mask airway (cLMA) is inserted, or with an introducer tool according to the manufacturer’s recommendations. Although cLMA insertion success on the first attempt with this technique is high, the PLMA insertion success rate is lower than that of the cLMA (91% vs 82%). Downfolded of the epiglottis during device insertion, the distal cuff folded over backwards, impaction at the back of the mouth, and failure of the distal cuff to reach its correct position in the hypopharynx can cause failed and/or delayed insertion with these techniques [1–3].

Many techniques have been described to facilitate LMA insertion, and these techniques have improved insertion conditions and insertion success rate [4–9]. It was first reported by Lee that the laryngoscope can improve the placement of LMA in an adult [10]. Then, direct laryngoscopy alone or laryngoscope-assisted guided techniques were used for this purpose [4, 8, 11–14]. These methods do have theoretical disadvantages, such as haemodynamic and airway stimulation and pharyngeal or oesophageal trauma [4, 8].

The C-MAC® videolaryngoscope (Karl Storz, Tuttlingen, Germany) provides several advantages for airway management because it improves the laryngeal view without the need for aligning all axes and ensures high-quality images with stable hemodynamic status during laryngoscopy [15, 16]. Recently, the Glidescope™/gastric tube-guided technique was used to facilitate difficult PLMA positioning [17].

We hypothesized that the C-MAC® videolaryngoscope-guided PLMA insertion technique would provide a better success rate for PLMA insertion than that of the standard digital technique. The purpose of the present study was to compare the insertion success rate and insertion conditions of the PLMA between the videolaryngoscope-guided insertion technique and the standard digital technique.

## Methods

The prospective, randomized controlled study adheres to CONSORT guideline. This study was performed after approval of the local ethics committee (Malatya Clinical Research Ethics Committee, 2019/36, February 20, 2019) and written informed consent from the patients.

The study was registered prior to patient enrolment at clinicaltrials.gov (identifier: NCT03852589, principal investigator: Ulku Ozgul, date of registration: February 22, 2019). We enrolled 119 patients with an American Society of Anesthesiologists (ASA) physical status of I-II, who were aged between 18 and 65 years and were scheduled for elective surgery in the supine position under general anaesthesia using the PLMA for airway management between March 2019 and April 2019. Patients with increased aspiration risk, body mass index > 35 kg/m^2^, a known or predicted difficult airway (Mallampati score > 2 or mouth opening < 3 cm), a disease related to the cervical spine, pre-existing sore throat or hoarseness or those with anticipated difficult airway were excluded.

Before anesthesia induction, patients were premedicated with 0.02 mg/kg i.v. midazolam. In the operating room, standard anesthetic monitoring was applied with electrocardiogram, non-invasive blood pressure, and peripheral oxygen saturation monitoring. All patients underwent a standard general anesthesia technique without the use of neuromuscular blocking agent after 3 min of preoxygenation with a face mask. Induction of anesthesia was carried out with remifentanil 2 μg/kg over 60 s and propofol 2 mg/kg mixed with 40 mg lidocaine over 30 s. The patients were ventilated with a facemask until conditions were suitable for PLMA insertion (loss of eyelash reflex, jaw relaxation, and the absence of movement). Additional boluses of 0.5 mg/kg i.v. propofol were given as required until an adequate level of anesthesia was achieved for PLMA placement. The PLMA was checked for leaks, and the back surface was lubricated with a water-soluble gel and sixty seconds after induction, the PLMA was inserted by an experienced anesthetist. Patients were not aware of the groups allocated. The data during anesthesia and postoperative period were collected by blinded observers.

Using a web-based randomization generation sequence from random allocation rule, the patients were randomly divided into two groups of 60 each [18]. The C-MAC® videolaryngoscope-guided insertion group was named Group V, and the standard digital insertion group was named Group D. All interventions were performed using a midline approach on patients in the sniffing position with the cuff fully deflated. The size of the PLMA was determined according to the patient’s weight: size 3 for ≤50 kg, size 4 for 50–70 kg, size 5 for 70–100 kg.

The videolaryngoscope-guided technique was performed a C-MAC® videolaryngoscope with D-Blade as follows. Under gentle videolaryngoscope guidance, the epiglottis was lifted, and the PLMA was advanced until the tip of the distal cuff reached the oesophagus inlet.

The standard digital technique was performed according to the manufacturer’s instructions [19]. In Group D, the PLMA was pressed with the index finger and forwarded around the palatopharyngeal curve until the resistance was felt.

After the PLMA was inserted, the cuff was inflated with air based on the amount of air proposed by the manufacturing company. With the cuff manometer (VBM Medizintechnik, Sulz, Germany), the maximum pressure was set to 60 cmH_2_O. Effective ventilation was confirmed using chest expansion and square wave capnography. Then, it was fixed according to the manufacturer’s recommended [19].

A well-lubricated gastric tube (14 French) was inserted along the drainage tube. Correct gastric tube placement was evaluated by fluid suction or air injected by epigastric stethoscopy.

A maximum of three attempts were allowed for the insertion of PLMA. If insertion failed after these attempts, alternative techniques were used, and the patient was excluded. Upon failed PLMA passage into the pharynx, PLMA malposition (air leakage despite cuff inflation), or ineffective ventilation (maximum expired tidal volume < 6 ml/kg), the trial was defined as failed insertion.

After successful PLMA insertion, anesthesia was maintained with sevoflurane 1.5 to 2%, using a 50:50 mixture of oxygen and air and remifentanil infusion (0.05–0.2 μg/kg /min). Patients were ventilated in synchronized intermittent mandatory ventilation mode until the end of the operation.

The heart rate, mean arterial blood pressure and peripheric oxygen saturation levels were recorded prior to anesthesia induction (t0); immediately after induction (t1); immediately after insertion of the PLMA (t2); and at 3 min (t3), 5 min (t4), and 10 min (t5) after PLMA insertion.

The incidence of postoperative airway morbidity during PLMA insertion and anesthesia, such as desaturation, airway obstruction, coughing, laryngospasm, bronchospasm, and trauma of the mouth, lip and tongue, were recorded. Any visible blood staining on the videolaryngoscope blade or PLMA was documented at removal.

At the end of the operation, the PLMA was removed when patients were able to sufficient spontaneous respiration and obey comments. After patients were taken to the recovery unit, sore throat, dysphagia, and dysphonia were noted within the postoperative period of 1 to 24 h. Symptoms were graded by the patient as mild, moderate or severe. Trained observers collected the data at 1 h and 24 h postoperatively.

The primary outcome was the first-attempt insertion success rate. The number of insertion attempts was recorded.

Secondary outcomes were insertion time, oropharyngeal leak pressure (OLP), leak volume, fibreoptic bronchoscopic view, peak inspiratory pressure, haemodynamic changes and postoperative airway morbidity.

When the OLP was measured, the pressure-limiting valve was set to 40 cmH_2_O, the expiratory valve of the circle system was fixed at a gas flow of 3 L/min and the ventilator was placed in manual mode. For measuring OLP, the ventilator pressure gauge and spirometer were used and defined as the point at which the steady state of airway pressure was reached. Oropharyngeal leak pressure was detected by both an audible noise that could be heard over the mouth and manometric stability; the leak was equilibrated with fresh gas flow [20].

Leak volume was evaluated by the difference between the inspiratory and expiratory tidal volumes and obtained from the anesthesia machine’s spirometry measurements during mechanical ventilation. The leak volume was measured three times, and its average was recorded. Peak inspiratory pressures were noted.

Insertion time was recorded as the time from picking up the device (or videolaryngoscope blade) until the appearance of the first square capnography wave.

The anatomical position of the PLMA was assessed with a fibreoptic bronchoscope (11302BD2, diameter 3.7 mm; length 65 cm; Karl Storz, Tuttlingen, Germany) by a blinded observer. The scoring used was that described by Brimacombe and Berry in our study as follows: 4 = only vocal cords visible; 3 = vocal cords plus posterior epiglottis visible; 2 = vocal cords plus anterior epiglottis visible; and 1 = vocal cords invisible [21].

The insertion of the orogastric tube was graded using a subjective scale of 1–3: 1 = easy; 2 = difficult; 3 = impossible to insert the device.

The minimum sample size required to detect a significant difference of the first attempt between the groups required at least 56 in each group (112 in total), considering type I error (alpha) of 0.05, power (1-beta) of 0.8, effect size of 0.6 and a two-sided alternative hypothesis [4].

The data were expressed as the mean (standard deviation, SD), median (min-max) or frequency with percentage depending upon the overall variable distribution. Normality was assessed using the Shapiro-Wilk test. The qualitative data were analysed with Pearson’s chi-square test, Yate’s corrected chi-square test and Fisher’s exact test where appropriate. The quantitative data were analysed by independent samples t-test and Mann Whitney U test as appropriate. The normally distributed data for repeated observations were compared by repeated measures analysis of variance (rANOVA) accompanied by Bonferroni test. *P* < 0.05 values were considered significant. IBM SPSS Statistics for Windows, Version 25.0. Armonk, NY: IBM Corp. was used for statistical analyses.

## Results

**Fig. 1:**
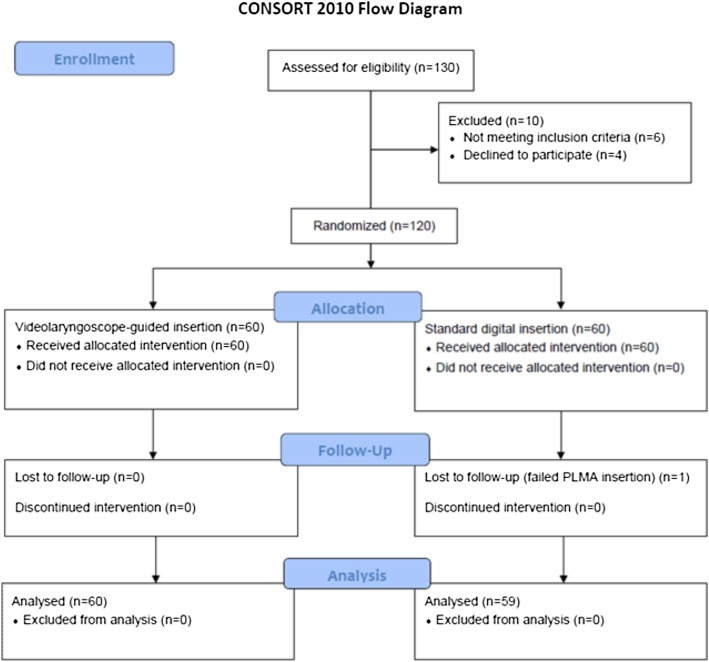
Consort Diagram

One hundred and twenty patients were recruited for the study. One patient was excluded from the study due to failed PLMA insertion in Group D. A total of 119 patients were included in the statistical analysis (Fig. 1).

Patient characteristics were shown in Table 1. The first-attempt success rate was higher in Group V than in Group D (*p* = 0.029). The effect size values with 95% confidence interval were 0.19 (0.01–0.36) for the first and second attempts, 0.09 (− 0.08–0.27) for the first and third attempts, and not computed for the second and third attempts by the groups, respectively.

**Table 1:** Patients characteristics. Data expressed as frequency (%), mean ± SD

Variables	Group V (*n* = 60)	Group D (*n* = 59)	*P value*
Age (y)	38.13 ± 11.45	38.16 ± 13.58	0.987
Weight (kg)	75.33 ± 11.84	74.22 ± 13.03	0.627
Height (cm)	170.45 ± 9.29	167.93 ± 8.42	0.125
BMI (kg/m^2^)	25.95 ± 3.8	26.31 ± 4.02	0.618
Gender (M:F)	37 /23	29 /30	0.190
ASA (I:II)	43 / 17	41 /18	0.797
Mallampati (I:II)	53 / 7	47 /12	0.200
Duration of Anesthesia (min)	96.83 ± 27.31	87.13 ± 33.73	0.087
Size of PLMA (3/4/5)	2/44/14	6/32/21	0.071

Group V, videolaryngoscope-guided group; Group D, standard digital group; M, male; F, female; BMI, body mass index; ASA, American Society of Anesthesiologists; PLMA, Proseal™ laryngeal mask airway

The fibreoptic position scores were better in Group V than in Group D (*p* < 0.001). In Group V, the fibreoptic view was found to be Brimacombe’s grade 4 in 45 patients (75%) and grade 3 in 15 patients (25%). There were no grade 2 or grade 1 in the patients. In Group D, the fibreoptic view was found to be Brimacombe’s grade 4 in 15 patients (25.4%), grade 3 in 16 patients (27.1%), grade 2 in 22 patients (37.2%) and grade 1 in 6 patients (10.1%).

The PLMA insertion time was longer in Group V than in Group D (*p* < 0.001). There were no differences in oropharyngeal leak pressures between the groups. The peak inspiratory pressure was lower in Group V than in Group D (*p* = 0.004). Orogastric tube insertion was more successful in Group V compared to Group D (p < 0.001) (Table 2).

**Table 2:** Comparative data of PLMA insertions. Data are mean ± standard deviation, frequencies or median (min-max)

Variables	Group V (n = 60)	Group D (n = 59)	*P value*	*Effect size with 95% C.I.*
Insertion success (n)				
First attempt	60	53 (89.8%)		0.19 (0.01–0.36)^a^
Second attempt	0	4 (6.7%)	0.029	0.09 (−0.08–0.27)^b^
Third attempt	0	1 (1.6%)		NaN^c^
Overall	60/60 (100%)	59/60 (98.3%)		
Insertion time (sec)	38.36 ± 6.44	28.59 ± 9.44	< 0.001	
Oropharyngeal leak pressure (cmH_2_O)	30.28 ± 8.3	29.86 ± 6.91	0.764	
Leak volume (L)	0.13 ± 0.15	0.19 ± 0.26	0.143	
Fibreoptic score	4 (3–4)	3 (1–4)	< 0.001	
Peak inspiratuar pressure (cmH_2_O)	11.21 ± 2.94	13.01 ± 3.73	0.004	
Orogastric tube insertion				
easy / difficult / impossible	60/0/0	46/11/2	< 0.001	

Group V, videolaryngoscope-guided group; Group D, standard digital group; ^a^ First and second attempts by the groups (2 × 2 crossstab); ^b^ First and third attempts by the groups (2 × 2 crossstab); ^c^ second and third attempts by the groups (2 × 2 crossstab) can not be computed

The haemodynamic parameters (heart rate and mean arterial pressure) were similar at all measurement times between the two groups (Table 3). The HR and MA*P* values in immediately after induction and at 1 min, 3 min, 5 min and 10 min after intubation were lower compared with the baseline values between the two groups.

**Table 3:** Hemodynamic parametres. Data are mean ± standard deviation

Variables	Group V (*n* = 60)	Group D (*n* = 59)	*P* value
Heart rate (beat/min)			
Baseline	78.18 ± 12.47	81.33 ± 12.59	0.17
After induction immediately	71.26 ± 9.85	71.25 ± 10.39	0.995
After intubation 1 min	68.41 ± 9.17	69.11 ± 10.28	0.695
After intubation 3 min	67.5 ± 9.01	69.13 ± 9.22	0.33
After intubation 5 min	67.63 ± 8.91	70.64 ± 10.22	0.089
After intubation 10 min	66.75 ± 10.67	70.2 ± 9.74	0.068
Mean Arterial Pressure (mmHg)			
Baseline	99.35 ± 10.09	101.15 ± 13.38	0.408
After induction immediately	69.55 ± 9.51	70.47 ± 10.51	0.616
After intubation 1 min	68.98 ± 8.78	67.71 ± 5.67	0.351
After intubation 3 min	68.8 ± 7.7	68.69 ± 6.94	0.938
After intubation 5 min	71.28 ± 8.84	71.38 ± 9.17	0.949
After intubation 10 min	72.63 ± 9.2	74.27 ± 9.56	0.343

Group V, videolaryngoscope-guided group; Group D, standard digital group

Postoperative airway morbidity was similar between the two groups. Postoperative sore throat was observed in 7 patients in Group V and 4 patients in Group D at 1 h and in 2 patients in Group V and 2 patients in Group D at 24 h, and there were no differences between the groups in the incidence of sore throat. Postoperative dysphagia developed in two patients in Group V and two patients in Group D, and there was no difference between the groups. Postoperative dysphonia was not observed in any patients. The visible blood on the PLMA after tube removal was observed in two patients in Group V and 5 patients in Group D, and there was no statistically significant difference (Table 4).

**Table 4:** Complications after removal of PLMA. Data are presented as frequencies

Variables	Group V (*n* = 60)	Group D (*n* = 59)	*P value*
Sore throat mild/moderate/severe (n)			
Postoperative 1 h	7/0/0	4/0/0	0.55
Postoperative 24 h	2/0/0	2/0/0	0.99
Dysphagia mild/moderate/severe (n)			
Postoperative 1 h	2/0/0	2/0/0	0.99
Postoperative 24 h	0/0/0	0/0/0	
Dysphonia mild/moderate/severe (n)			
Postoperative 1 h	0/0/0	0/0/0	
Postoperative 24 h	0/0/0	0/0/0	
Visible blood on PLMA (n)	2	5	0.27

Group V, videolaryngoscope-guided group; Group D, standard digital group

## Discussion

We found that the first-attempt success rate of the PLMA with the videolaryngoscope-guided insertion technique was superior. Additionally, fibreoptic scoring, orogastric tube placement success and peak inspiratory pressure were better in the videolaryngoscope-guided technique than in the standard digital technique.

It was reported that second-generation SADs (i-gel, PLMA, LMA Supreme) have reliable first-time placement, high seal pressure, separation of gastrointestinal and respiratory tracts and are recommended to intubation fail for airway rescue as well by Difficult Airway Society guideline [22]. Successful placement is most likely on the first attempt. Repeated attempts at inserting a SAD increases the probability of airway trauma and may delay the decision to accept failure. Many studies demonstrated that in cases of failed conventional insertion of SADs, PLMA manufacturer’s introducer, 90° rotational technique, laryngoscope/videolaryngoscope-assisted or catheter-assisted techniques had the high success rate [9]. So, we designed the study to compare with the videolaryngoscope asisted and digital technique.

There are contradictory results of LMA insertion using direct laryngoscopy or laryngoscope-assisted guided techniques. Kim et al. showed that the rate of success at the first attempt was similar between standard digital and laryngoscope-guided insertion [14]. However, many other studies that used different LMA types indicated that the laryngoscope-guided insertion technique is more successful than were the digital or rotational techniques [8, 11, 14, 15, 23]. Additionally, the laryngoscope-guided, gum elastic bougie-guided technique was superior to the digital and introducer technique [4]. The success rate on the first attempt was also higher in the videolaryngoscope-guided technique in our study. A possible reason for the higher insertion success rate in the videolaryngoscope-guided technique was the ability to direct the distal cuff around the back of the mouth and into the hypopharynx, which improves the functional and anatomical optimization. The success rate in the present study for the standard digital technique was similar to that in previous studies [2–4].

At the suitable depth of anesthesia, spontaneous breathing may be easily inhibited by opioids and hypnotic drugs without neuromuscular blockers. No using neuromuscular blocking agent prevents the undesirable side effects of these agents, such as prolonged neuromuscular block, and may lead to the need of neuromuscular antagonist [9, 24]. So, we did not prefer a neuromuscular blocking agent.

Laryngeal mask airway placement can assesed using fibreoptic laryngoscopy [13]. Positioning can be confirmed by fibreoptic evaluation, on which vocal cords were clearly seen, often with the posterior part of epiglottis visible (but not the tip) and with the cuff optimally placed on the midline. Fibreoptic scoring was used in previous studies; however, different results were reported. Campbell et al. found that 91.5% of direct laryngoscopy patients had an ideal LMA insertion position; however, an ideal fibreoptic position was observed in 42% of patients in the standard digital group. Our results are consistent with those of Campbell et al. [13]. Videolaryngoscopy provides visualization of the epiglottis and can prevent downfolding of the epiglottis, distal cuff misplacement and backward folding, as well as proximal LMA cuff displacement during LMA placement. Therefore, video laryngoscopy may improve insertion conditions and prevent airway gas leaks, airway obstruction and impaired gas exchange [25].

The oropharyngeal leak test is usually administered to quantify the seal with the airway for using an LMA [19]. The double-cuff design of the PLMA provides an excellent sealing effect for positive pressure ventilation compared to cLMA [2, 3]. Kim et al. reported that the OLP (21.4 ± 8.6 cmH_2_O) was higher in the laryngoscope-guided technique that used cLMA [13]. Our OLP value was 30 cmH_2_O in the videolaryngoscope-guided technique and was different from their finding. A possible reason for the higher OLP was that the PLMA device was used in our study. The results of our OLP value were similar to that in studies that used the PLMA [4].

It was reported that LMA placement using the standard digital technique prevents airway trauma and avoids haemodynamic changes [2, 26]. In our study, haemodynamic parameters and incidence of postoperative airway morbidity were similar in both groups. The reason for these results may be the use of C-MAC® videolaryngoscopy, the incorporation of gently lifting the epiglottis. Many studies have indicated that videolaryngoscopy leads to fewer haemodynamic responses than does direct laryngoscopy during endotracheal intubation [17, 27]. The C-MAC® videolaryngoscope may be also less traumatic than direct laryngoscopy [28]. Additionally, the studies with laryngoscope-guided insertion of an LMA did not present significant differences in haemodynamic parameters compared to the standard digital technique [8, 14, 23].

The insertion time was longer (by approximately 10 s) in the videolaryngoscope-guided insertion group than in the standard digital insertion group (38 vs 28 s) in the present study. Several studies determined that the insertion time with direct laryngoscopy or laryngoscope-assisted guided techniques was longer than the time required for the standard digital technique [7, 14]. Our results are also similar to these. The result may be related to the videolaryngoscope-guided insertion group requiring extra time for laryngoscope insertion. However, the difference is not clinically important, as emphasized in previous studies [7, 28].

Correct positioning of the PLMA can be detected by correct orogastric tube insertion. Smooth passage of a drainage tube into the stomach shows that the distal cuff of the PLMA is not folded, and the lumen is aligned with the oesophagus [25, 28]. When the PLMA does not achieve an ideal position, it allows venting of air during positive pressure ventilation. Orogastric tube insertion success in our study showed superiority of the videolaryngoscope-guided insertion technique over the standard digital technique. This finding may prove that PLMA insertion significantly improves the ideal positioning.

There are some limitations in the present study. First, all interventions were by the same, more experience anesthetist, and the results may not be applicable to less experienced practitioners. Second, skill acquisition of the C-MAC videolaryngoscope requires a brief period of learning and regular practice. Third, all patients had Mallampati scores I or II, so our results may not conform to patients who had potentially difficult airways. Finally, the anesthetist who performed PLMA insertion was not blinded, which may lead to bias. However, other observers who collected data were blinded.

## Conclusion

The standard digital insertion technique has successful insertion rate with easy, time saving, cheap, be used everywhere and simple training. Videolaryngoscope-guided insertion technique can be a help in case of difficult positioning of a PLMA and can improve the PLMA performance in some conditions. We suggest that the videolaryngoscope-guided technique may be a useful technique if the digital technique fails.

## Data Availability

The datasets used during the current study are available from the corresponding author on reasonable request.

## Abbrevations

ASA: American Society of Anesthesiologists
cLMA: classic™ laryngeal mask airway
OLP: oropharyngeal leak pressure
PLMA: Proseal™ laryngeal mask airway
